# Fernblock, a Nutriceutical with Photoprotective Properties and Potential Preventive Agent for Skin Photoaging and Photoinduced Skin Cancers

**DOI:** 10.3390/ijms12128466

**Published:** 2011-11-29

**Authors:** Salvador Gonzalez, Yolanda Gilaberte, Neena Philips, Angeles Juarranz

**Affiliations:** 1 Dermatology Service, Memorial Sloan-Kettering Cancer Center, New York, NY 10065, USA; 2 Dermatology Service, Ramon y Cajal Hospital, Madrid 28034, Spain; 3 Dermatology Service, Hospital San Jorge, Huesca 22004, Spain; E-Mail: ygilaberte@salud.aragon.es; 4 School of Natural Sciences, University College, Fairleigh Dickinson University, Teaneck, NJ 07666, USA; E-Mail: nphilips@fdu.edu; 5 Biology Department, Sciences School, Universidad Autónoma de Madrid, Madrid 28049, Spain; E-Mail: angeles.juarranz@uam.es

**Keywords:** *Polypodium leucotomos* extract, photoprotection, oral sunscreen, antioxidant, photoaging, skin cancer

## Abstract

Many phytochemicals are endowed with photoprotective properties, *i.e.*, the capability to prevent the harmful effects of excessive exposure to ultraviolet (UV) light. These effects include photoaging and skin cancer, and immunosuppression. Photoprotection is endowed through two major modes of action: UV absorption or reflection/scattering; and tissue repair post-exposure. We and others have uncovered the photoprotective properties of an extract of the fern *Polypodium leucotomos* (commercial name Fernblock). Fernblock is an all-natural antioxidant extract, administered both topically (on the skin) or orally. It inhibits generation of reactive oxygen species (ROS) production induced by UV including superoxide anion. It also prevents damage to the DNA, inhibits UV-induced AP1 and NF-κB, and protects endogenous skin natural antioxidant systems, *i.e.*, CAT, GSH, and GSSR. Its photoprotective effects at a cellular level include a marked decrease of UV-mediated cellular apoptosis and necrosis and a profound inhibition of extracellular matrix remodeling. These molecular and cellular effects translate into long-term inhibition of photoaging and carcinogenesis that, together with its lack of toxicity, postulate its use as a novel-generation photoprotective nutriceutical of phytochemical origin.

## 1. Introduction

Modern approaches to the use of natural products in medicine include the concentration of the active principles in the form of lipophilic or hydrophilic extracts, which are then delivered systemically (in the form of pills or syrups) or topically, in the form of poultices, ointments or creams. *Polypodium leucotomos* is a tropical fern that belongs to the *Polypodiaceae* family [1]. This and other ferns belonging to the same family have value in traditional, natural medicine in South America, and were commonly used to make poultices to treat psoriasis and other skin disorders, although with variable success [2]. However, their promise as potential natural treatments for several skin disorders has generated an interest in several pharmaceutical companies and research groups. These efforts have resulted in the production of a concentrated hydrophilic extract of the leaves of *Polypodium leucotomos* (commercial name Fernblock), which is endowed with photoprotective properties. These have been shown in several cellular, animal and human models and studies. These studies constitute a growing body of knowledge that supports its wide use in the prevention of photoaging and suggests its efficacy as a preventive measure against UV-induced cancers.

## 2. UV Radiation and the Skin: Regular Foes and Occasional Friends

Ultraviolet (UV) photons are endowed with wavelengths 100–400 nm. For study purposes, the Commission Internationale de L’ Éclairage (CIE) and other international organisms classify UV radiation into three major subtypes, depending on the wavelength: UVA, (315–400 nm), UVB (280–315 nm) and UVC (100–280 nm). UVA, UVB and UVC photons can damage the skin. However, UVC photons are blocked in the upper layers of the atmosphere, thus they do not pose a health problem except in those regions where the ozone layer is weakened. It is necessary to note that very limited exposure to sun radiation (hence UV photons) is not only beneficial, but also required for human health. Indeed, UV photons catalyze the conversion of vitamin D in the skin [3]. However, unprotected or excessive exposure to UV photons has catastrophic consequences on the health of the skin, e.g., DNA damage, inflammation and immunosuppression. Most of these are due to the generation of ROS (Reactive Oxygen Species). With regards to DNA damage, some of the molecular components of the DNA absorb UVB wavelengths. UVB induce the formation of cyclobutane pyrimidine dimers (CPD, especially thymine-thymine) and pyrimidine-pyrimidone dimers [4,5]. These UV byproducts cause immunosuppression [6] and carcinogenesis [7]. UV-mediated isomerization of trans urocanic acid (trans-UCA) induces immunosupression [8]. In addition, UVA photons can mutate the DNA directly through ROS-induced oxidative damage. ROS produced this way indirectly promote the generation of 8-hydroxy-2′-deoxyguanosine (8-oxo-dG) [9] which is considered a bona fide marker of DNA oxidative damage [10].

UV photons also induce skin erythema, vasodilation and elevated blood flow, activation of endothelial cells and expression of pro-inflammatory markers, which causes leukocyte recruitment of immune cell infiltration [11–13]. Amplification of the immune response also occurs due to apoptotic bodies accumulated in the skin caused by ROS-induced cell death [14,15]. An apparently contradictory effect is the induction of UV-mediated immunosuppression and immunological tolerance. UV induces a marked decrease in the numbers of Epidermal Langerhans cells (eLCs), which causes Th1 clonal anergy [16].

The molecular basis of most of these effects is related to oxidation and local generation of ROS formation in the skin [17,18]. Continued exposure to UV photons overwhelms the endogenous antioxidant mechanisms causing local oxidative stress. ROS induce oxidative damage through several mechanisms: one is the peroxidation of fatty acids integral to the plasma and nuclear membranes, promoting membrane flip-flop and contributing to cellular apoptosis; ROS can also induce DNA damage (see previous paragraph); finally, they can oxidize proteins, which correlates with accelerated aging [19].

In summary, ROS are one of the major causes of environment-mediated skin aging (“photoaging”).

## 3. Fernblock: A Multi-Pronged Approach to Photoprotection

### 3.1. Formulation and Biological Disposition

Fernblock is but one of a newer generation of natural photoprotective preparations that can be used either systemically (oral intake) or topically, by application on the skin. A major part of its photoprotective properties rely on its antioxidant properties due to its high phenolics content. Fernblock’s phenolics moiety include mostly benzoates and cinnamates, e.g., caffeic and ferulic acids [20]. Caffeic acid inhibits UV-induced peroxide and nitric oxide (NO) formation, whereas ferulic acid quenches UV photons [21,22]. Interestingly, PL efficiently scavenges superoxide anion, similar to superoxide dismutase [23,24]. These have been shown to inhibit skin erythema induced by sun exposure [25]. Fernblock also contains different types of biological acidic molecules, e.g., quinic, shikimic, glucuronic, malic coumaric, and vanillic acids; and monosaccharides (mainly fructose and glucose) [26].

*In vitro* and *in vivo* studies on the pharmacology and biological disposition of Fernblock have revealed that its toxicity is negligible even at high doses [26], which further substantiates its use as an oral, systemic photoprotector. It is also readily absorbed through the skin, thus being employed as a direct local photoprotector [27]. The photoprotective dose in healthy humans is 7.5 mg/kg [28,29]. Topically, erythema was inhibited using 0.1% (weight/volume) PLE [24].

### 3.2. Molecular, Cellular and Clinical Evidence of the Photoprotective Properties of Fernblock

The extract that constitutes the basis of Fernblock (PL) has been analyzed thoroughly to reveal the molecular mechanisms through which it endows photoprotection. A summary of these effects is shown in Table 1.

#### 3.2.1. DNA Photoprotection

Administered in oral form, PL inhibits DNA damage and mutagenesis. PL prevented the UV-induced accumulation of cyclobutane pyrimidine dimers in a mouse model [30] and also in a small-sample clinical study with healthy human volunteers [29]. This may be due to an improved function of the DNA repair systems, perhaps due to decreased oxidative damage [40]. It also reduced basal oxidative damage, as shown by the reduction in the percentage of 8-hydroxy-2′-deoxyguanosine-positive cells [30]. These effects likely result in the observed improvement. Finally, another small-sample clinical study has revealed that PL decreases UVA-dependent mitochondrial DNA damage as evidenced by decreased “common deletion” (CD) [31], which is a mitochondrial marker of chronic UVA radiation in fibroblasts and keratinocytes [41].

#### 3.2.2. Anti-Inflammatory Activity

There is also evidence of the anti-inflammatory properties of PL. PL prevented sunburn and erythema in UV-treated human skin [24,27,29], and also in psoralen-UVA (PUVA)-based therapy [28], which is often used in the treatment of psoriasis, vitiligo and other inflammatory skin conditions [42,43]. The molecular basis of its anti-inflammatory properties can be explained in terms of its ability to suppress the expression of pro-inflammatory molecules and markers, e.g., TNFalpha and iNOS [32], among others. This is consistent with its ability to block the transcriptional activation of AP-1 and NF-κB induced by UV radiation [32]. Importantly, PL inhibits UV-induced expression of COX-2 [30]. This is important as COX-2 induces the synthesis of PGI2, which is a potent inducer of vasodilation and inhibits platelet aggregation [44]. Together, these effects account for decreased leukocyte extravasation and presence of mast cells in the irradiated area [29]. PL also inhibits apoptosis and cell death of the cells that populate the layers of the skin, e.g., fibroblasts and keratinocytes [32,33], which would otherwise result in a local inflammatory response.

#### 3.2.3. Inhibition of Photoimmunosuppression

UV radiation induces skin immunosuppression. One mechanism is by elimination of skin dendritic cells (Langerhans cells). These professional APC (antigen-presenting cells) are crucial mediators of the skin immune response, and UV promotes their disappearance from skin [45]. Another mechanism is through activation of suppressor T cells by *cis*-urocanic acid. *cis*-urocanic acid results from the UV-mediated isomerization of *trans*-urocanic acid, which is a natural sunscreen found mainly in the *stratum corneum* of the skin [46].

Experiments using rodent models have revealed that PL blocks UVB-induced immunosuppression [36]. PL prevents the elimination of Langerhans cells induced by UV irradiation during direct UV exposure [35], or PUVA-therapy [28]. It also inhibits the isomerization of *trans*-urocanic acid into the *cis* form and prevents further photo-induced breakdown and degradation in a cell-free system [34]. Finally, analysis of the blood and skin of mice treated orally with PL and subsequently irradiated with UV revealed that PL decreased the levels of oxidized glutathione and increased the activity of the enzyme catalase, suggesting a positive systemic effect in the antioxidant systems, particularly in skin [35].

#### 3.2.4. Prevention of Matrix Remodeling and Other Cellular Effects

PL exhibits a strong anti-aging effect. It prevents the morphological effects associated with increased oxidative stress, which include a dramatic disorganization of the microfilaments and loss of cell-matrix and cell-cell anchorage points [38]. Additional anti-aging effects of PL include inhibition of the expression and activation of several matrix metalloproteases and increased expression of an endogenous metalloprotease inhibitor, TIMP. Other molecules that are down-regulated during the onset of photoaging, such as elastin, collagen and TGF-β, are markedly stimulated by PL [39].

UV radiation also damages the cellular membranes by inducing lipid peroxidation [47]. PL counters this effect and thus prevents subsequent membrane damage [37].

#### 3.2.5. Anti-Skin Tumor Capability

Several studies have documented the anti-tumor properties of different fern extracts, including PL [48,49]. For example, in a hairless albino mice model, PL blocked tumor formation in the skin as a result of exposure to UVB [50]. A number of features support the anti-carcinogenic capability of PL. For one, it blocks ROS formation and their effects (see above). Similarly, its anti-mutagenic properties also protect from immortalizing mutations leading to carcinogenesis [30]. As outlined above, PL inhibits the increase of COX-2 induced by UV radiation [30], which is also involved in carcinogenesis [51–53]. Also, PL induced activation of the tumor suppressor p53 [30]. Finally, it is interesting to note that PL complemented the effect of ascorbate in limiting melanoma cell growth and their ability to remodel the extracellular matrix through, among other effects, increasing the expression of the metalloprotease inhibitor TIMP-1 [54].

### 3.3. Potential Use of Fernblock in the Treatment of Pathological Skin Conditions

#### 3.3.1. Idiopathic Photodermatosis

These lesions include clinical conditions that emanate from exposure of the skin to normal sunlight. Some examples include polymorphic light eruption (PLE), solar urticaria, chronic actinic dermatitis and actinic prurigo [55]. A very recent study has addressed the potential of PL to counter the occurrence of PLE [56]. Despite taking into account the limitations of the patient cohort and the open nature of the trial, this study suggests that PL has a positive effect in the treatment of PLE, which may be extended to other idiopathic photodermatoses.

#### 3.3.2. Vitiligo

One of the most efficient methods to treat vitiligo vulgaris is narrow band (311–312 nm) UVB phototherapy, which stimulates melanocyte reservoirs to counter depigmentation. A double blind, placebo-controlled study has showed that the conjoined use of PL with narrow-band UVB (NB-UVB) increases the repigmentation of the head and neck area of vitiligo patients [57]. Together with its beneficial effect on PUVA-therapy [28,58], these studies suggest that PL may be a beneficial, general use adjuvant in phototherapy protocols.

## 4. Fernblock: A Road to (Present and Future) Photoprotection

Fernblock exhibits a wide array of beneficial effects and displays no significant toxicity or allergenic properties. Its dual route of administration, *i.e.*, topical and oral, suggest that it is efficient as a preventer of UV-induced damage (taken orally before exposure), as a protector during exposure (both orally and topically) and also may contribute to the healing and regeneration of the skin that is required post-exposure. These regenerative properties also underlie its potential as an anti-aging and anti-cancer tool. Most of its beneficial effects are related to its antioxidant and ROS scavenging capability, but its ability to prevent apoptosis and block the improper ECM rearrangements that occur during oxidative damage suggest that its profile may extend beyond skin care and be useful as a systemic antioxidant tool. Further research will be aimed to study its effect in other parameters related to aging, e.g., telomere length and telomerase activity, *etc*.

## Figures and Tables

**Table 1:** Summary of the photoprotective effects of PL.

	Effect	Reference
**DNA**	Prevents formation of cyclobutane pyrimidine dimmers;	[30]
	Inhibits basal oxidative damage (marker, 8-hydroxy-2′-deoxyguanosine);	[30]
	Blocks damage to mitochondrial DNA.	[31]

**Inflammation**	Prevents sunburn and erythema;	[24,27,29]
	Blocks TNFα, iNOS, AP-1, NF-κB;	[32]
	Inhibits expression of COX-2;	[30]
	Prevents apoptosis.	[32,33]

**Immunosuppression**	Blocks t-UCA isomerization;	[34]
	Enhances natural antioxidant systems;	[35]
	Prevents depletion of Langerhans cells;	[27,29,35]
	Preserves LC function.	[36]

**Other cellular effects**	Blocks lipid peroxidation;	[37]
	Inhibits cytoskeletal disarray and MMP expression and activation.	[38,39]
